# Sex differences and determinants of coronary microvascular function in asymptomatic adults with type 2 diabetes

**DOI:** 10.1016/j.jocmr.2024.101132

**Published:** 2024-12-06

**Authors:** Jian L. Yeo, Abhishek Dattani, Joanna M. Bilak, Alice L. Wood, Lavanya Athithan, Aparna Deshpande, Anvesha Singh, J.Ranjit Arnold, Emer M. Brady, David Adlam, John D. Biglands, Peter Kellman, Hui Xue, Thomas Yates, Melanie J. Davies, Gaurav S. Gulsin, Gerry P. McCann

**Affiliations:** a Department of Cardiovascular Sciences, University of Leicester and the National Institute for Health and Care Research (NIHR) Leicester Biomedical Research Centre, Glenfield Hospital, Leicester, United Kingdom; b Radiology, University Hospitals of Leicester NHS Trust, Leicester, United Kingdom; c NIHR Leeds Biomedical Research Centre and Medical Physics and Engineering, Leeds Teaching Hospitals NHS Trust, Leeds, United Kingdom; d National Heart, Lung, and Blood Institute, National Institutes of Health, Department of Health and Human Services, Bethesda, Maryland, USA; e Diabetes Research Centre, University of Leicester and the NIHR Leicester Biomedical Research Centre, Leicester General Hospital, Leicester, United Kingdom

**Keywords:** Type 2 diabetes, Coronary microvascular dysfunction, Myocardial perfusion, Cardiovascular magnetic resonance

## Abstract

**Background:**

Coronary microvascular dysfunction (CMD) is a significant complication in type 2 diabetes (T2D) and may be more common in women. We aimed to evaluate the sex differences and sex-specific clinical determinants of CMD in adults with T2D without prevalent cardiovascular disease.

**Methods:**

Single center pooled analysis of four prospective studies comparing asymptomatic people with T2D and controls. All subjects underwent comprehensive cardiovascular phenotyping with myocardial perfusion reserve (MPR) quantified with perfusion cardiovascular magnetic resonance (CMR). Participants with silent coronary disease were excluded. Multivariable linear regression was performed to identify determinants of MPR with an interaction term for sex.

**Results:**

Four hundred and seventy-nine T2D (age 57 ± 11 years, 42% [202/479] women) were compared with 116 controls (age 53 ± 11 years, 41% [48/116] women). Men with T2D, but not women, demonstrated worse systolic function and higher extracellular volume fraction than controls. MPR was significantly lower in T2D than controls (women, 2.6 ± 0.9 vs 3.3 ± 1.0, p < 0.001; men, 3.1 ± 0.9 vs 3.5 ± 1.0, p = 0.004), and lower in women than men with T2D (p < 0.001). More women than men with T2D had MPR <2.5 (46% [79/202] vs 26% [64/277], p < 0.001). There was a significant interaction between sex and body mass index (BMI) for MPR (p interaction <0.001). Following adjustment for clinical risk factors, inverse association with MPR were BMI in women (β = −0.17, p = 0.045) and systolic blood pressure in men (β = −0.14, p = 0.049).

**Conclusion:**

Among asymptomatic adults with T2D, women had a greater prevalence of CMD than men. Risk factors modestly but significantly associated with CMD in asymptomatic people with T2D were BMI among women and systolic blood pressure among men.

## Graphical abstract

**Figure:**
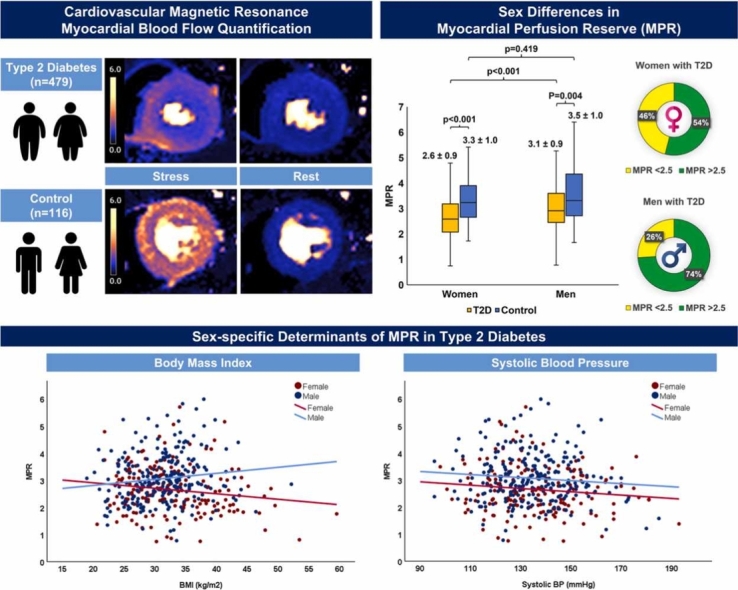

## 1 Background

Microvascular complications are a major source of morbidity and mortality in type 2 diabetes (T2D), with approximately 20% prevalence globally [1]. The pathophysiology of microvascular disease is complex, mediated by a cascade of pathological processes across multiple vascular beds, including chronic inflammation, oxidative and osmotic stress, and vascular endothelial dysfunction [2]. In people without obstructive epicardial coronary artery disease, coronary microvascular dysfunction (CMD) can lead to angina and exercise intolerance. Importantly, CMD has been implicated in the development of diastolic dysfunction and heart failure with preserved ejection fraction (HFpEF), a common phenotype among those with T2D and obesity [3].

It is recognized that women, defined as female sex assigned at birth, have a predisposition to CMD and have poorer outcomes compared to men [4]. A substantial proportion of women who present with angina have non-obstructive coronary arteries, and their symptoms may be attributed to CMD [5]. Furthermore, physiological differences in myocardial blood flow (MBF) between women and men have been previously reported among healthy volunteers [6], highlighting the importance of a sex-stratified evaluation of CMD. However, no studies to date have assessed sex-specific differences of CMD in asymptomatic subjects with T2D.

CMD can be assessed non-invasively by calculation of myocardial perfusion reserve (MPR), which is the ratio of stress to rest MBF. MPR has been shown to be lower in people with T2D compared to non-diabetic controls, even in the absence of prevalent cardiovascular disease [7]. Furthermore, we have previously reported an independent and inverse association between MPR and peak aerobic exercise capacity in asymptomatic adults with T2D [7]. In people with symptomatic HFpEF, MPR was lower compared to controls (1.7 ± 0.8 vs 2.2 ± 0.8, p = 0.001) and was independently predictive of adverse outcomes during a median follow-up of 3.1 years [8]. These findings suggest that CMD is an important contributor to exercise intolerance in T2D, occurs before development of clinical symptoms, and is associated with poor prognosis. However, the determinants of MPR among asymptomatic people with T2D, and whether they differ by sex, remain unclear.

The aims of this study are to (1) evaluate the sex differences in MPR and (2) identify the sex-specific clinical determinants of MPR, in a multi-ethnic cohort with T2D without prevalent cardiovascular disease. We hypothesized that MPR would be lower in women with T2D and that determinants of MPR would differ by sex.

## 2 Methods

### 2.1 Study population

This was a pooled analysis of individual patient data from four prospective studies at a single center, evaluating the impact of T2D on cardiovascular structure and function [9], [10], [11], [12] with additional healthy controls included from a study assessing spontaneous coronary artery dissection [13]. Participants were recruited from primary and specialist care services in Leicestershire, UK, with support of the National Institute for Health Research East Midlands Clinical Research Network. Participants were aged ≥18 to ≤75 years with a diagnosis of T2D and no prior history, signs, or symptoms of cardiovascular disease (including symptomatic coronary, peripheral, or cerebrovascular disease, valvular heart disease, arrhythmias, or heart failure). Exclusion criteria were diagnosis of type 1 diabetes mellitus, estimated glomerular filtration rate <30 mL/min/1.73 m^2^, or absolute contraindication to cardiovascular magnetic resonance (CMR) imaging. Participants with common co-morbidities associated with T2D, such as obesity, treated hypertension, and dyslipidemia, were included. Sex- and ethnicity-matched non-diabetic volunteers were included for comparison. Ethical approval for each study was granted by the United Kingdom National Research Ethics Service, and they were conducted according to the Declaration of Helsinki (references 17/WM/0192, 15/WM/0222, 13/WM/0311, 09/H0407/9, and 14/EM/0056). All participants provided written informed consent before any testing.

### 2.2 General examinations

Demographics, medical history, and anthropometric measurements were collected. The presence of hypertension and/or hypercholesterolemia was obtained from history or by prescribed medication to treat these conditions. A fasting blood sample was collected for biochemical profiling including full blood count, liver function, renal function, lipid profile, and glycosylated hemoglobin (HbA1c). Samples were collected on the same day as the imaging procedures and analyzed in an accredited National Health Service pathology lab at the University Hospitals of Leicester. Blood pressure (BP) was measured in clinic as previously described [12].

### 2.3 Transthoracic echocardiography

Transthoracic echocardiography was performed and reported by accredited operators using an iE33b system with X5-1 transducer (Philips Medical Systems, Best, The Netherlands) as per the American Society of Echocardiography guidelines [14]. Early (E) and late (A) diastolic mitral inflow velocities and early diastolic mitral annular velocities (e′) were assessed by Doppler echocardiography.

### 2.4 Cardiovascular magnetic resonance imaging

CMR was performed using a standardized protocol on either a 1.5T or 3T Siemens scanners (Erlangen, Germany) as previously described [7]. After localizers, steady-state free precession cine images were acquired in the four-, three-, and two-chamber long-axes views. MBF image acquisition was performed using a saturation recovery gradient-echo pulse sequence with signal intensity versus time curves converted to concentration curves using a linear signal response to contrast agent with Fermi-constrained deconvolution (at 1.5T) [15] or dual-sequence gradient-echo method with inline automated reconstruction and post-processing (at 3T) [16]. Participants abstained from caffeine for 24 h before CMR. Perfusion images were acquired following vasodilator stress with adenosine (140–210 mg/kg/min) infusion for 3–5 min. Adequate hemodynamic response to adenosine was determined objectively by heart rate increase of ≥10% and/or systolic BP reduction of ≥10 mmHg, accompanied by self-reported mild symptoms such as chest tightness, tachypnoea, or flushing. At peak stress, a gadolinium-based contrast agent ((Gadobutrol, Bayer Healthcare, Berlin, Germany, 0.04 mmol/kg or Gadoteric acid, Guerbet, Aulnay-sous-Bois, France, 0.075 mmol/kg for dual sequence) was injected followed by a 20 mL bolus of normal saline, at a rate of 5 mL/s. Perfusion images were acquired at three short-axis LV planes (basal, mid-ventricular, and apical). Rest imaging was performed approximately 10 min after stress.

In between stress and rest perfusion imaging, a stack of short-axis cine slices was obtained with coverage of the entire left ventricle (LV). Late gadolinium enhancement (LGE) images were acquired at least 5 min after rest perfusion for assessment of silent myocardial infarct and focal myocardial fibrosis. LGE images were acquired in the same positions as long- and short-axes cine images using a phase-sensitive inversion recovery reconstruction sequence. Native T1 mapping was performed using the Modified Look-Locker sequence before stress perfusion and post-contrast T1 maps were performed after LGE imaging in a subset of the cohort.

### 2.5 CMR image analysis

CMR images were analyzed using cvi42 (Version 5.10.1, Circle Cardiovascular Imaging, Calgary, Alberta, Canada) by one of two trained observers blinded to participant demographic and clinical details as previously described [7]. LV mass to end-diastolic volume ratio (LVM/V) was calculated as a marker of LV concentric remodeling. LV strain and strain rate are presented as absolute values, where lower values indicate worse myocardial mechanics [17].

LGE images were assessed visually for subendocardial or transmural LGE indicative of previous silent myocardial infarction was recorded. Perfusion images were first assessed qualitatively (G.P.M.) for regional perfusion defects indicative of ischemia due to epicardial coronary disease as per clinical standards [18]. To minimize the impact of epicardial disease on assessment of coronary microvascular function, participants with regional ischemia in a coronary distribution or myocardial infarction on LGE imaging were excluded from quantitative MBF analysis. Global MPR was derived as the ratio of average stress to rest blood flow across all myocardial segments, without correction for rate-pressure product. We have chosen a cut-off value of MPR <2.5 to define CMD, based on previous reports of optimal cut-offs ranging between 2 and 2.5 [19], [20], [21]. Myocardial extracellular volume fraction (ECV), a surrogate marker of diffuse interstitial fibrosis, was calculated from pre- and post-contrast T1 maps [22].

### 2.6 Statistical analysis

Normality was assessed visually using histograms and Q-Q plots. Continuous data were expressed as mean (standard deviation) if normally distributed or median (25th–75th percentile range) if not. For imaging parameters, T2D and controls were compared with adjustment for age and ethnicity by analysis of covariance and values expressed as estimated marginal means (95% confidence interval). Categorical variables were presented as N (%) and compared using chi-square test.

Univariable associations between clinical and biochemical measures with MPR were assessed. Multivariable linear regression was performed to identify the independent determinants of MPR. Clinical variables recognized to be associated with myocardial perfusion from the literature were a priori entered into the multivariable model and included: age, sex, ethnicity, smoking status, body mass index (BMI), systolic BP, diabetes duration, number of diabetes medications, creatinine, HbA1c, and low-density lipoprotein. Multicollinearity was avoided by ensuring a variance inflation factor of <2.5. Regression analysis was performed first in the entire T2D cohort and then stratified by sex to determine the sex-specific clinical determinants. Given different sex-related associations with BMI and BP, interaction between sex and BMI and systolic BP in the adjusted model was assessed.

Associations between MPR and imaging measures of LV remodeling (LVM/V), fibrosis (ECV), systolic (global longitudinal strain [GLS], global circumferential strain [GCS], LV ejection fraction [LVEF]) and diastolic function (E/A, E/e′) in women and men were performed after adjusting for age, ethnicity, smoking status, and adding significant clinical predictors from initial regression analysis. All beta (β) values are presented as standardized coefficients. Statistical analysis was performed using Statistical Package for Social Services version 28.0 (SPSS Inc. Chicago, Illinois, USA). A p value <0.05 was considered statistically significant.

## 3 Results

### 3.1 Clinical characteristics

**Fig. 1:**
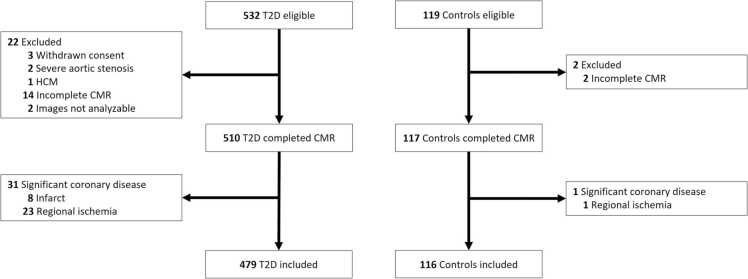
Study flowchart. *CMR* cardiovascular magnetic resonance, *HCM* hypertrophic cardiomyopathy, *T2D* type 2 diabetes

The study flowchart is presented in Fig. 1. Five hundred and thirty-two subjects with T2D and 119 controls were enrolled. Four hundred and seventy-nine T2D (42% [202/479] women) and 116 controls (41% [48/116] women) were available for analysis following removal of those that met exclusion criteria or had occult coronary disease (Additional File 1: Fig. S1). Sex-stratified clinical characteristics of study participants are presented in Table 1. Compared to controls, men with T2D were older and had a higher prevalence of smoking history. Both groups were matched for ethnicity. Women and men with T2D had higher BMI, diastolic BP, and heart rate whereas systolic BP was higher only in women compared to controls. Anti-hypertensive and lipid-lowering medication use were also greater among those with T2D. People with T2D had higher triglycerides but lower cholesterol indices.

**Table 1:** Baseline clinical characteristics in T2D and controls.

	Women			Men		
	T2D (n = 202)	Controls (n = 48)	p value	T2D (n = 277)	Controls (n = 68)	p value
Age, y	55.7±11.1	54.4±10.0	0.451	57.9±11.2	51.2±12.0	**<0.001**
Ethnicity			0.283			0.826
White	139 (69)	38 (79)		196 (71)	50 (73)	
Asian	59 (29)	10 (21)		74 (27)	17 (25)	
Other	4 (2)	0		7 (2)	1 (2)	
Smoking status			0.346			**0.029**
Never smoked	137 (68)	34 (71)		135 (48)	44 (65)	
Ex-smoker	43 (21)	12 (25)		106 (38)	21 (31)	
Current smoker	22 (11)	2 (4)		36 (13)	3 (4)	
BMI, kg/m^2^	33.4±7.1	24.7±3.2	**<0.001**	31.4±5.3	26.2±3.7	**<0.001**
Duration of diabetes, y	7 (3–11)	-	-	7 (3–12)	-	-
Hypertension	90 (45)	2 (4)	**<0.001**	163 (59)	8 (12)	**<0.001**
Hypercholesterolemia	126 (62)	4 (8)	**<0.001**	182 (66)	7 (10)	**<0.001**
Systolic BP, mmHg	135.7±17.8	124.6±19.2	**<0.001**	135.2±15.5	132.6±18.8	0.229
Diastolic BP, mmHg	82.2±10.3	78.5±10.0	**0.023**	83.9±8.7	81.3±9.4	**0.035**
Heart rate, bpm	78.8±11.9	65.6±9.9	**<0.001**	74.8±12.3	64.5±11.2	**<0.001**
Medication				57.9±11.2	51.2±12.0	
ACE inhibitor/ARB	75 (39)	2 (5)	**<0.001**			**<0.001**
Beta blocker	11 (6)	1 (2)	0.356	196 (71)	50 (73)	**0.040**
Calcium channel blocker	40 (21)	1 (2)	**0.004**	74 (27)	17 (25)	**0.001**
Statin	121 (61)	4 (8)	**<0.001**	7 (2)	1 (2)	**<0.001**
Diet-controlled diabetes	24 (12)	NA	NA			NA
Biguanide	158 (78)	NA	NA	135 (48)	44 (65)	NA
SGLT-2 inhibitor	37 (18)	NA	NA	106 (38)	21 (31)	NA
GLP-1 receptor agonist	25 (12)	NA	NA	36 (13)	3 (4)	NA
Sulphonylurea	33 (16)	NA	NA	31.4±5.3	26.2±3.7	NA
DPP-4 inhibitor	25 (12)	NA	NA	7 (3–12)	-	NA
Insulin	24 (12)	NA	NA	163 (59)	8 (12)	NA
Biochemistry						
Hemoglobin, g/dL	134.4±13.2	134.5±11.0	0.983	148.8±12.8	151.3±10.5	0.218
Estimated GFR, mL/min/1.73 m^2^	93.3±15.4	91.8±19.9	0.595	93.4±16.2	97.2±15.7	0.123
Fasting glucose, mmol/L	8.0±2.4	5.0±0.4	**<0.001**	8.1±2.4	5.2±0.7	**<0.001**
HbA1c, mmol/mol	57.3±13.3	35.7±3.4	**<0.001**	56.6±12.7	36.2±3.5	**<0.001**
HbA1c, %	7.4±1.2	5.4±0.3	**<0.001**	7.3±1.2	5.5±0.3	**<0.001**
Cholesterol:HDL ratio	3.5±1.3	3.0±0.8	**0.007**	3.6±1.0	3.5±1.0	0.360
LDL, mmol/L	2.5±0.8	3.1±0.9	**<0.001**	2.2±0.9	3.3±0.9	**<0.001**
Triglycerides, mmol/L	1.6 (1.2–2.2)	1.0 (0.7–1.4)	**<0.001**	1.6 (1.1–2.4)	1.0 (0.8–1.5)	**<0.001**

Values are mean ± SD, n (%), or median (IQR).

Bold indicates p<0.05

*ACE* angiotensin-converting enzyme, *ARB* angiotensin receptor blocker, *BMI* body mass index, *BP* blood pressure, *DPP-4* dipeptidyl peptidase-4, *GFR* glomerular filtration rate, *GLP-1* glucagon-like peptide-1, *HbA1c* glycated hemoglobin, *HDL* high-density lipoprotein, *LDL* low-density lipoprotein, *SGLT-2* sodium-glucose co-transporter-2, *T2D* type 2 diabetes, *SD* standard deviation, *IQR* interquartile range, *NA* not applicable

### 3.2 Cardiac structure and function

Sex-stratified imaging parameters are presented in Table 2. Compared to controls, people with T2D had higher LVM/V, driven primarily by increased LV mass in women and reduced LV end-diastolic volume in men. Those with T2D also had worse diastolic function demonstrated by lower E/A and higher E/e′ ratios. Men with T2D demonstrated lower GLS, higher ECV, and lower left atrial maximal volume than male controls, whereas LVEF was higher only in women with T2D. GCS was not different between T2D and controls. A higher proportion of men had non-ischemic LGE compared to women, but there was no difference between T2D and controls in both sexes.

**Table 2:** Echocardiographic and CMR parameters in T2D and controls.

	Women					Men				
	n	T2D (n = 202)	n	Controls (n = 48)	p value^a^	n	T2D (n = 277)	n	Controls (n = 68)	p value^a^
Echocardiography										
E/A ratio	162	0.94 (0.91–0.97)	38	1.07 (1.01–1.13)	**<0.001**	239	0.92 (0.89–0.95)	47	1.08 (1.02–1.15)	**<0.001**
E/e′ ratio	159	9.4 (9.0–9.8)	38	8.3 (7.5–9.1)	**0.025**	233	8.5 (8.2–8.7)	47	7.5 (7.0–8.0)	**<0.001**
CMR										
LVM, g	202	100.3 (97.5–103.1)	48	92.0 (86.3–97.7)	**0.011**	277	128.6 (125.9–131.2)	68	129.6 (124.1–135.1)	0.740
LVM/height, g/m	202	62.1 (60.6–63.7)	48	56.1 (52.9–59.4)	**0.001**	277	73.5 (72.1–75.0)	68	73.6 (70.6–76.5)	0.990
LVEDV, mL	202	121.1 (117.7–124.4)	48	124.8 (117.9–131.6)	0.343	277	149.1 (145.4–152.9)	68	163.0 (155.3–170.7)	**0.002**
LVEDV/height, mL/m	202	74.9 (73.0–76.8)	48	76.1 (72.1–80.0)	0.611	277	85.2 (83.3–87.2)	68	92.5 (88.4–96.6)	**0.002**
LVM/V, g/mL	202	0.84 (0.83–0.86)	48	0.74 (0.71–0.78)	**<0.001**	277	0.88 (0.86–0.90)	68	0.81 (0.77–0.84)	**<0.001**
LV ejection fraction, %	202	69.2 (68.3–70.0)	48	67.2 (65.4–68.9)	**0.043**	277	65.0 (64.3–65.8)	68	64.7 (63.2–66.3)	0.726
Global longitudinal strain, %	202	17.2 (16.8–17.5)	47	17.6 (16.9–18.3)	0.248	277	16.1 (15.8–16.3)	68	16.7 (16.2–17.3)	**0.037**
Global circumferential strain, %	201	20.2 (19.8–20.5)	47	20.3 (19.6–21.0)	0.776	276	18.6 (18.3–18.9)	68	18.5 (17.9–19.1)	0.805
Longitudinal PEDSR, s^−1^	197	0.81 (0.78–0.84)	46	0.82 (0.76–0.88)	0.754	268	0.69 (0.67–0.71)	68	0.69 (0.65–0.73)	0.938
Circumferential PEDSR, s^−1^	197	1.04 (1.01–1.07)	46	1.08 (1.02–1.15)	0.232	263	0.89 (0.86–0.91)	68	0.95 (0.90–0.99)	**0.019**
ECV fraction, %	156	27.7 (27.3–28.1)	18	27.3 (26.1–28.4)	0.292	230	26.5 (26.1–26.8)	35	25.1 (24.2–25.9)	**0.005**
LA volume, mL	196	62.4 (59.7–65.2)	45	65.5 (59.7–71.3)	0.346	274	68.1 (65.5–70.7)	68	74.4 (69.1–79.8)	**0.037**
LA volume/height, mL/m	196	38.7 (37.0–40.3)	45	39.9 (36.4–43.4)	0.527	274	38.9 (37.5–40.4)	68	42.3 (39.3–45.2)	0.051
Non-ischemic LGE		12 (6)		4 (8)	0.648		54 (20)		10 (15)	0.583
Myocardial blood flow										
Rest MBF, mL/min/g	176	1.07 (1.01–1.14)	47	0.96 (0.84–1.08)	0.113	252	0.82 (0.78–0.85)	66	0.67 (0.60 to 0.75)	**<0.001**
Rest MBF corrected for RPP, mL/min/g	176	1.03 (0.96–1.10)	47	1.23 (1.09–1.36	**0.010**	252	0.82 (0.78–0.85)	66	0.83 (0.76–0.90)	0.751
Stress MBF, mL/min/g	176	2.67 (2.53–2.81)	48	3.00 (2.74–3.26)	**0.028**	250	2.44 (2.33–2.54)	66	2.34 (2.13–2.55)	0.417
MPR	173	2.65 (2.51–2.79)	47	3.30 (3.04–3.57)	**<0.001**	246	3.08 (2.96–3.20)	66	3.46 (3.23–3.69)	**0.004**
MPR <2.5		79 (46)		8 (17)	**<0.001**		64 (26)		13 (20)	0.290

Values are displayed as estimated marginal means (95% confidence interval) adjusted for age and ethnicity, or n (%)

Bold indicates p<0.05

*E/A* early to late diastolic mitral inflow velocity ratio, *E/e′* early diastolic mitral inflow to annular velocity ratio, *ECV* extracellular volume fraction, *LA* left atrium, *LGE* late gadolinium enhancement, *LVEDV* left ventricular end-diastolic volume, *LVM* left ventricular mass, *LVM/V* left ventricular mass to end-diastolic volume ratio, *MBF* myocardial blood flow, *MPR* myocardial perfusion reserve, *PEDSR* peak early diastolic strain rate, *RPP* rate-pressure product, *CMR* cardiovascular magnetic resonance, *T2D* type 2 diabetes, *LV* left ventricular

a p values adjusted for age and ethnicity

### 3.3 CMR myocardial perfusion

**Fig. 2:**
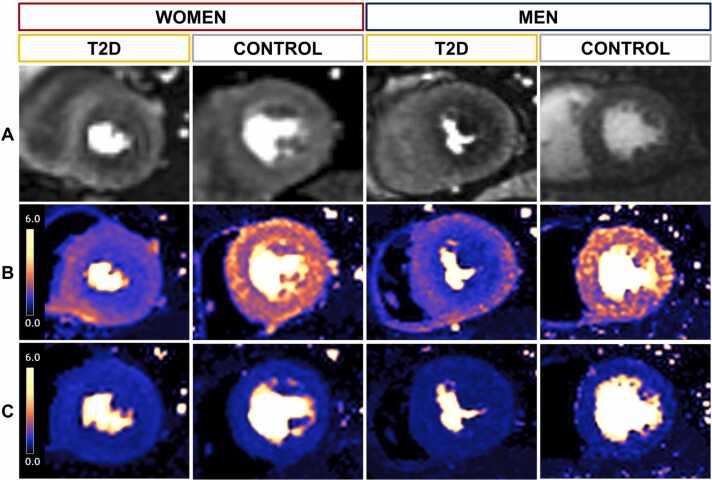
Representative images displaying pixel-wise myocardial perfusion maps in T2D and controls (A) first-pass perfusion images, (B) stress, and (C) rest perfusion maps of the left ventricular mid short-axis slice in T2D and controls. Women: the example with T2D is that of a 62-year-old, BMI 35 kg/m^2^, BP 145/77 mmHg, stress MBF 1.40 mL/kg/min, rest MBF 0.71 mL/kg/min, and MPR 1.97. The non-diabetic control is a 65-year-old, BMI 24 kg/m^2^, BP 121/80 mmHg, stress MBF 2.39 mL/kg/min, rest MBF 0.69 mL/kg/min, and MPR 3.46. Men: the example with T2D is that of a 55-year-old, BMI 34 kg/m^2^, BP 168/99 mmHg, stress MBF 1.53 mL/kg/min, rest MBF 0.56 mL/kg/min, MPR 2.73. The non-diabetic control is a 64-year-old, BMI 32 kg/m^2^, BP 160/99 mmHg, stress MBF 2.21 mL/kg/min, rest MBF 0.59 mL/kg/min, MPR 3.75. Note in both participants with T2D, the global stress MBF is lower (less orange) than for controls and with particularly reduced perfusion toward the subendocardium, which typifies CMD. *T2D* type 2 diabetes, *BMI* body mass index,

**Fig. 3:**
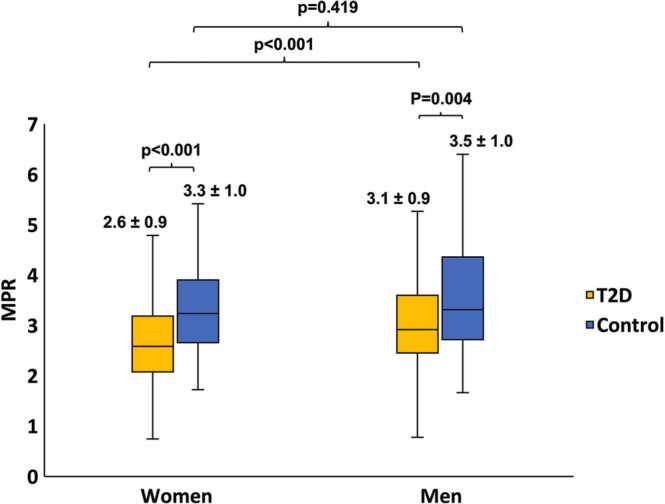
MPR in women and men with and without T2D. Women and men with T2D had lower MPR compared to non-diabetic controls. Among those with T2D, women had lower MPR compared to men, but this difference is not seen between non-diabetic controls. *MPR* myocardial perfusion reserve, *T2D* type 2 diabetes

Representative images of myocardial perfusion maps are presented in Fig. 2. Compared to controls, women with T2D had lower stress MBF while men with T2D had higher rest MBF (not when rate-pressure product corrected), with resulting MPR lower in both sexes. MPR values measured at 1.5T and 3T CMR field strengths were not significantly different in T2D (2.91 ± 1.03 vs 2.88 ± 0.84, p = 0.479). In controls, there was no difference in MPR between women and men (3.3 ± 1.0 vs 3.5 ± 1.0, respectively, p = 0.419) but in T2D, MPR was lower in women (2.6 ± 0.9 vs 3.1 ± 0.9, p < 0.001) (Fig. 3). In T2D, a significantly greater proportion of women compared to men had MPR <2.5 (46% [79/202] vs 26% [64/277], p < 0.001).

Among those with T2D, 14% (29/202) women and 11% (31/277) men were missing MPR data due to either patient-related (contraindication to adenosine, declined stress) or technical (contrast extravasation, perfusion sequence failure) reasons. Comparisons between those with missing and available MPR data are presented in Additional File 1: Table S1.

### 3.4 Associations between MPR and clinical risk factors in T2D

The univariable and multivariable regression analyses of participants with T2D, for the total cohort and stratified by sex, are presented in Table 3. In the total cohort of T2D, MPR was inversely associated with female sex and systolic BP in univariable and multivariable analyses after adjustments for clinical risk factors.

**Table 3:** Associations between MPR and clinical risk factors in people with T2D.

	All T2D				Women with T2D				Men with T2D			
	Univariable		Multivariable		Univariable		Multivariable		Univariable		Multivariable	
	β	p value	β	p value	β	p value	β	p value	β	p value	β	p value
Clinical and biochemical variables												
Age	−0.06	0.242	−0.07	0.237	−0.07	0.386	−0.17	0.072	−0.10	0.136	−0.01	0.951
Sex (female)	−0.23	**<0.001**	−0.24	**<0.001**	-	-	-	-	-	-	-	-
Ethnicity (White)	0.01	0.830	0.04	0.479	−0.06	0.458	0.03	0.763	0.05	0.445	0.07	0.354
Never smoked	−0.04	0.371	−0.002	0.973	0.09	0.232	0.09	0.292	−0.06	0.389	−0.07	0.290
BMI	−0.03	0.518	−0.01	0.918	−0.16	**0.040**	−0.17	**0.045**	0.13	**0.043**	0.14	0.067
Systolic BP	−0.12	**0.018**	−0.11	**0.040**	−0.12	0.106	−0.11	0.168	−0.10	0.136	−0.14	**0.049**
Diabetes duration	−0.05	0.304	−0.003	0.967	0.06	0.404	0.09	0.383	−0.14	**0.033**	−0.06	0.485
Number of diabetes medication	−0.05	0.317	−0.02	0.793	0.06	0.420	0.05	0.626	−0.10	0.131	−0.06	0.448
Creatinine	0.07	0.129	−0.02	0.760	0.06	0.419	0.09	0.248	−0.10	0.124	−0.07	0.316
HbA1c	−0.05	0.341	−0.04	0.423	−0.02	0.755	−0.09	0.306	−0.05	0.470	−0.03	0.672
LDL	−0.01	0.906	0.01	0.852	−0.01	0.870	0.003	0.971	0.04	0.520	0.03	0.641
									
R^2^			0.072				0.074				0.062	

Data are presented as standardized coefficients (β).

Bold indicates p<0.05.

*BMI* body mass index, *BP* blood pressure, *LDL* low-density lipoprotein, *T2D* type 2 diabetes, *HbA1c* glycated hemoglobin

When stratified by sex, MPR was associated with BMI in women, and BMI and diabetes duration in men, on univariable analysis. Following multivariable adjustments, MPR was independently associated with BMI only in women and systolic BP only in men.

### 3.5 Sex interactions with BMI and systolic BP

**Fig. 4:**
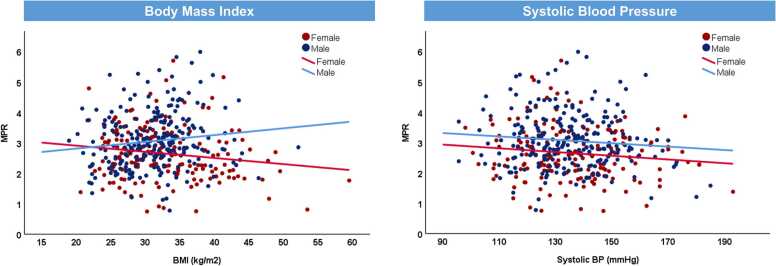
Multivariable association between myocardial perfusion reserve (MPR) with (A) BMI and (B) systolic BP in people with T2D, stratified by sex. There was a significant interaction between sex and BMI for MPR (p interaction < 0.001). There was no interaction between sex and systolic BP for MPR (p interaction = 0.888). *BMI* body mass index, *BP* blood pressure, *T2D* type 2 diabetes

The interaction term for sex and BMI was significant (p < 0.001) indicating lower MPR with increasing BMI in women but not men (Fig. 4A). The interaction term for sex and systolic BP was not significant (p = 0.888) (Fig. 4B).

### 3.6 Association between MPR and markers of LV remodeling, fibrosis, and function in T2D

The associations between imaging markers of structure and function and MPR are presented in Additional File 1: Table S2. There was no association between LVM/V or ECV with MPR. Following multivariable adjustments, GCS and LVEF were inversely and independently associated with MPR. For diastolic function, only E/A showed a positive association with MPR in women.

## 4 Discussion

This is the first study to comprehensively describe sex differences and determinants of coronary microvascular function in asymptomatic adults with T2D. We found that in the absence of significant regional ischemia, women but not men, with T2D had lower stress MBF compared to non-diabetic controls, resulting in lower MPR. The proportion of CMD in T2D was almost double in women compared to men, highlighting that early subclinical microvascular dysfunction is especially prevalent among women with T2D. Female sex and systolic BP were independently and inversely associated with MPR. When stratified by sex, the risk factors inversely associated with MPR were BMI in women and systolic BP in men. The interaction between men and women and MPR was significant for BMI but not significant for BP. These findings may have important implications for interventions to prevent or treat CMD in women and men with T2D.

Our study has several strengths. Our T2D cohort is the largest to prospectively undertake detailed phenotyping with CMR including quantitative perfusion imaging. We have recruited a multi-ethnic cohort of participants with an excellent proportion represented by women. We rigorously excluded people with symptoms or history of cardiovascular disease, therefore representing the asymptomatic population of interest who are at risk of developing subsequent cardiovascular disease.

### 4.1 Sex differences in myocardial blood flow

Sex differences in MBF have been widely reported. Previous positron emission tomography (PET) [23] and CMR [6], [24] studies have shown that healthy women have higher absolute rest (even when corrected for rate-pressure product) and stress MBF compared to men, but similar MPR. This phenomenon is also seen in a large symptomatic patient cohort (n = 1218, 67% [813/1218] women, approximately 30% [363/1218] diabetes) with non-obstructive coronary disease referred clinically for PET [25]. The reason for why women have higher MBF than men is unclear, but may be due to underlying differences in resting vascular tone [26] and greater catecholamine surge during adenosine stress in women [23]. Due to the inherent sex differences in MBF, we sought to assess the effect of T2D on MBF and MPR by comparing people with T2D to non-diabetic controls, in men and women separately.

In a previous study using quantitative perfusion CMR, Sorenson et al. reported that people with T2D had higher rest and lower stress MBF, resulting in lower MPR, compared to controls, although they did not report data stratified by sex, which is a key strength of our analyses [27]. In this study, the lower MPR in T2D is driven by lower stress MBF but no significant change in rest MBF among women, whereas among men lower MPR is primarily due to higher rest MBF. In diabetic hearts, altered carbohydrate metabolism and reduced myocardial glucose uptake cause a shift from glucose utilization to fatty acid oxidation to generate energy [28], [29]. Indeed, impaired glucose utilization in diabetes is more marked in men than women [30]. Fatty acid oxidation consumes more oxygen [31] and a higher resting MBF may be an adaptive response to meet the increased myocardial oxygen demand. We speculate that the inability to increase rest MBF, coupled with a poorer hyperemic augmentation of MBF in response to adenosine seen in women, reflects a greater deleterious impact of T2D on endothelial and microvascular function. This may partly explain the higher relative risk of adverse outcomes associated with diabetes, including heart failure, in women compared to men [32], [33].

### 4.2 Associations between myocardial perfusion with clinical and biochemical measures

Systolic BP was significantly and inversely associated with MPR across the entire cohort of T2D, and when stratified by sex, was significant only in men. Furthermore, there was no sex interaction with systolic BP on MPR, with similar beta coefficients in multivariable analysis in both women and men. Although our T2D cohort had systolic BP largely within normal ranges, further BP reduction may be favorable as reported in a recent large meta-analysis [34], in part by improving coronary perfusion. However, clinical studies of the effect of BP reduction on MPR in people with T2D are lacking.

BMI was the only modifiable risk factor significantly associated with MPR in women in our study. Notably, the difference in BMI between T2D and controls was relatively greater in women than men. Visceral adipose tissue promotes inflammation through secretion of adipokines leading to vascular endothelial damage [35]. Accumulation of excess visceral adiposity is associated with a more detrimental effect on women than men, such as in the development of diabetes [36] and HFpEF [37]. Furthermore, sex differences in efficacy of weight-loss interventions have been reported. Women lost more weight with pharmacotherapy while men responded better with lifestyle (diet or exercise) interventions [38]. For example, in the STEP 2 trial (n = 1210, 50.9% [616/1210] female) where people with T2D were randomized to either semaglutide or placebo for 68 weeks, the odds ratio of achieving a weight loss of ≥5% and ≥10% with semaglutide 2.4 mg were 12.1 and 33.8, respectively, in women compared to 5.9 and 6.4 in men [39]. The effect of weight loss on MPR in T2D, either with diet or pharmacotherapy, has not been specifically studied in men or women only. One may speculate that weight loss may have a greater impact on CMD in women than men, but this is required to be tested in randomized trials.

A higher HbA1c has been previously shown to be associated with a greater risk of microvascular disease [40] and glycemic control optimization was linked to better LV systolic and diastolic function [41]. However, in our study, neither HbA1c nor other indicators of diabetes severity such as diabetes duration or number of diabetes medications were associated with MPR. This lack of association concurs with a previous report [42] and suggests that strict glycemic control per se may not have a significant impact on coronary microvascular function. Indeed, researchers have been exploring whether newer pharmacotherapy could improve coronary microvascular function. SGLT-2 inhibitors are now a key therapy for heart failure due to the benefits in reducing mortality and heart failure hospitalizations [43]. However, the SIMPLE trial, in which 90 people with T2D were randomized to either empagliflozin or placebo for 13 weeks, reported no short-term improvement in MPR [44].

### 4.3 Associations between myocardial perfusion with imaging markers of LV remodeling, fibrosis, and function

We did not find any association between MPR and LVM/V or ECV in both women and men. This finding reflects that CMD is present independent of altered myocardial structure in early diabetic cardiomyopathy. There were significant inverse associations between MPR with measures of systolic function which may seem counterintuitive. This result is also contrary to previous data from the Multi-Ethnic Study of Atherosclerosis cohort, comprising of asymptomatic people with and without diabetes, showing that lower myocardial perfusion is linked to lower LV systolic strain [45]. We speculate that the increased myocardial contractility is a physiological response to sympathetic hyperactivity secondary to the pro-inflammatory state in T2D [46], and that those with lower MPR have greater myocardial catecholamine release. There is a modest association between MPR with E/A in women only, and no association between MPR and E/e′, suggesting a weak but important signal indicating that the relationship between coronary microvascular function and diastolic function in asymptomatic T2D may be stronger in women than men.

## 5 Limitations

We acknowledge several limitations of our study. The cross-sectional, observational nature of our study precludes determination of causation or direction of causality. We did not perform anatomical assessment of coronaries with either invasive or CT coronary angiography. Primarily this is because we prospectively enrolled an asymptomatic cohort, in which our extensive array of non-invasive testing was deemed the most acceptable and ethically suitable approach for cardiovascular phenotyping. We have excluded those with regional ischemia on CMR perfusion imaging which has excellent diagnostic accuracy to detect functionally significant obstructive epicardial disease [18]. Furthermore, it is felt that the likelihood of silent obstructive three-vessel coronary disease in this cohort is extremely low, for several reasons. First, all included subjects were carefully screened to exclude those with symptoms of cardiovascular disease (angina or limiting dyspnea). Second, all participants underwent incremental cardiopulmonary exercise testing with electrocardiography (data not presented in this paper) and were excluded if positive for ischemia. Lastly, large-scale population-based studies indicate that the prevalence of three-vessel obstructive coronary artery disease in asymptomatic people is very rare; for example in the MIAMI Heart Study (N = 2359, 8.3% [196/2359] diabetes) the prevalence of any stenosis ≥50% on coronary computed tomography angiography was 5.9% (140/2359) and three vessels with stenoses ≥50% was 0.13% (3/2359) [47]. Although occult triple vessel disease cannot entirely be ruled out without invasive or CT coronary angiography, we are confident that our rigorous approach to screening and testing renders the likelihood of this to be negligible.

The use of two different acquisitions and analysis pathways to perform quantitative perfusion may have increased the variability of the absolute MBF value obtained. However, this would not impact MPR as it is a ratio of stress to rest MBF using the same approach at a per patient level. In our study, MPR values measured using the two different acquisitions were not significantly different in T2D (Additional File 1: Table S3). Similarly, two different contrast agents were used across the four studies and we cannot exclude that there may be minor differences in the MBF. However, similar numbers of women and men were included in each study and there is unlikely to be any systematic bias in MPR assessment between sexes. The multivariable model in our study had a modest R-squared value suggesting that a substantial proportion of the variability in MPR remains unexplained by the factors included in the current analysis and highlights the need to explore additional variables and alternative modeling approaches to better understand the sex-specific determinants of CMD in adults with T2D. We did not measure adiposity or muscle mass, which are better markers of body composition than BMI, and may explain the mechanism of CMD in this cohort. In our study, a higher ECV fraction is presumed to be due to increased myocardial fibrosis, but ECV may be raised for other reasons including myocardial edema, and is not a direct measure of tissue fibrosis.

## 6 Conclusions

In a large, multi-ethnic cohort of asymptomatic adults with T2D, we found that women had a greater prevalence of CMD than men. Female sex and systolic BP were inversely and independently associated with MPR. However, there was a significant sex interaction for BMI, only being significant in women. These findings may have important implications for prevention of CMD in T2D which should be assessed with trials of weight loss and BP control.

## Funding

This study was funded by the 10.13039/100006662NIHR Research Trainees Coordinating Centre through a Career Development Fellowship and Research Professorship Award (CDF 2014-07-045 and RP-2017-08-ST2-007 to G.P.M.), the 10.13039/501100000274British Heart Foundation (FS/16/47/32190 to G.S.G., FS/CRTF/20/24069 to A.D., FS/18/26/33501 to L.A., and PG/13/96/30608 to D.A.), the 10.13039/501100000265Medical Research Council through an Interdisciplinary Bridging Award, Novo Nordisk, NIHR rare disease translational collaboration, and BeatSCAD. A.S. is funded by an NIHR Advanced Fellowship (NIHR300867). J.R.A. is funded by an NIHR Clinical Scientist Fellowship. Study funders provided financial support but had no role in the study design (other than the external review process), data collection, data analysis, data interpretation, or writing of reports (including the current manuscript).

## Author contributions

E.M.B., T.Y., M.J.D., G.S.G., and G.P.M. were involved in the conception, design, and interpretation of the results. J.L.Y., A.D., J.M.B., A.L.W., and L.A. recruited study participants, supervised assessment visits, and clinical reviews. J.L.Y., J.D.B., P.K., and G.S.G. were involved in the analysis of data. J.L.Y. performed the statistical analysis and wrote the first draft of the manuscript. All authors edited, reviewed, and approved the final version of the manuscript. G.P.M. is the guarantor of this work and, as such, had full access to all the data in the study and takes responsibility for the integrity of the data and the accuracy of the data analysis.

## Ethics approval and consent

Ethical approval for each study was granted by the United Kingdom National Research Ethics Service, and they were conducted according to the Declaration of Helsinki (references 17/WM/0192, 15/WM/0222, 13/WM/0311, 09/H0407/9, and 14/EM/0056). All participants provided written informed consent prior to any testing.

## Consent for publication

Not applicable.

## Declaration of competing interests

The authors declare the following financial interests/personal relationships which may be considered as potential competing interests: Melanie Davies reports financial support was provided by UKRI Medical Research Council. Melanie Davies reports financial support was provided by Novo Nordisk Inc. Gerry McCann reports financial support was provided by National Institute for Health and Care Research. Gaurav Gulsin reports financial support was provided by British Heart Foundation. Abhishek Dattani reports financial support was provided by British Heart Foundation. Lavanya Athithan reports financial support was provided by British Heart Foundation. David Adlam reports financial support was provided by British Heart Foundation. Anvesha Singh reports financial support was provided by National Institute for Health and Care Research. J. Ranjit Arnold reports financial support was provided by National Institute for Health and Care Research. The other authors declare that they have no known competing financial interests or personal relationships that could have appeared to influence the work reported in this paper.

## Data Availability

The datasets used and/or analyzed during the current study are available from the corresponding author upon reasonable request.
