# Supplementary material for: Sex differences and determinants of coronary microvascular function in asymptomatic adults with type 2 diabetes
Source: J Cardiovasc Magn Reson. 2024 Dec 6;27(1):101132. doi: 10.1016/j.jocmr.2024.101132 (PMC11761338; doi:10.1016/j.jocmr.2024.101132)
Supplement: Supplementary file 1 — Supplementary material [file mmc1.docx]

# Additional Files

Table S1 Comparison between T2D participants with missing and available MPR data

|  | Women with T2D | | | Men with T2D | | |
| --- | --- | --- | --- | --- | --- | --- |
|  | MPR missing (n=29) | MPR available (n=173) | P value | MPR missing (n=31) | MPR available (n=246) | P value |
| Age, y | 57.3 ± 10.8 | 55.5 ± 11.2 | 0.419 | 58.6 ± 11.0 | 57.9 ± 11.2 | 0.738 |
| Ethnicity |  |  | 0.794 |  |  | 0.719 |
| White | 19 (66) | 120 (69) |  | 20 (65) | 176 (72) |  |
| Asian | 9 (31) | 50 (29) |  | 10 (32) | 64 (26) |  |
| Other | 1 (3) | 3 (2) |  | 1 (3) | 6 (2) |  |
| Smoking status |  |  | 0.757 |  |  | 0.382 |
| Never smoked | 18 (62) | 119 (69) |  | 16 (52) | 119 (48) |  |
| Ex-smoker | 7 (24) | 36 (21) |  | 9 (29) | 97 (39) |  |
| Current smoker | 4 (14) | 18 (10) |  | 6 (19) | 30 (12) |  |
| BMI, kg/m^2^ | 37.1 ± 7.9 | 32.8 ± 6.8 | **0.002** | 31.5 ± 5.7 | 31.4 ± 5.2 | 0.872 |
| Duration of diabetes, y | 8 (3 – 12) | 7 (3 – 11) | 0.749 | 7 (4 – 11) | 7 (3 – 12) | 0.985 |
| Hypertension | 13 (45) | 77 (45) | 0.974 | 23 (74) | 140 (57) | 0.065 |
| Hypercholesterolemia | 20 (69) | 106 (61) | 0.429 | 26 (84) | 156 (63) | **0.024** |
| Systolic BP, mmHg | 132.5 ± 18.8 | 136.2 ± 17.6 | 0.297 | 141.0 ± 16.0 | 134.5 ± 15.3 | **0.028** |
| Diastolic BP, mmHg | 81.3 ± 9.1 | 82.4 ± 10.5 | 0.583 | 85.4 ± 8.6 | 83.7 ± 8.7 | 0.292 |
| Heart rate, bpm | 77.7 ± 13.4 | 79.0 ± 11.7 | 0.580 | 73.5 ± 12.5 | 74.9 ± 12.3 | 0.546 |
| **Biochemistry** |  |  |  |  |  |  |
| Hemoglobin, g/dL | 136.3 ± 13.8 | 134.1 ± 13.1 | 0.410 | 145.9 ± 10.4 | 149.2 ± 13.0 | 0.193 |
| Estimated GFR, mL/min/1.73m^2^ | 96.8 ± 14.2 | 92.7 ± 15.5 | 0.186 | 92.0 ± 20.4 | 93.6 ± 15.7 | 0.606 |
| Fasting glucose, mmol/L | 8.1 ± 2.9 | 8.0 ± 2.2 | 0.778 | 8.9 ± 3.2 | 8.0 ± 2.2 | **0.043** |
| HbA1c, mmol/mol | 57.0 ± 16.3 | 57.3 ± 12.8 | 0.909 | 60.7 ± 17.2 | 56.1 ± 11.9 | 0.057 |
| HbA1c, % | 7.4 ± 1.5 | 7.4 ± 1.2 | 0.920 | 7.7 ± 1.6 | 7.3 ± 1.1 | 0.055 |
| Cholesterol:HDL ratio | 3.5 ± 0.9 | 3.5 ± 1.3 | 0.925 | 3.4 ± 1.0 | 3.7 ± 1.0 | 0.171 |
| Triglycerides, mmol/L | 1.7 (1.1 – 2.4) | 1.6 (1.2 – 2.2) | 0.820 | 1.5 (1.1 – 2.3) | 1.6 (1.1 – 2.4) | 0.706 |

See Table 1 for abbreviations.

Table S2 Association between MPR with cardiac structure and function in women and men with T2D

|  | Women with T2D | | | | Men with T2D | | | |
| --- | --- | --- | --- | --- | --- | --- | --- | --- |
|  | Univariable | | Multivariable^a^ | | Univariable | | Multivariable^a^ | |
|  | β | P value | β | P value | β | P value | β | P value |
| **LV remodelling and diffuse fibrosis** |  |  |  |  |  |  |  |  |
| LVM/V | 0.02 | 0.801 | 0.05 | 0.536 | -0.02 | 0.813 | 0.002 | 0.981 |
| ECV | 0.06 | 0.499 | 0.004 | 0.954 | -0.04 | 0.599 | 0.02 | 0.784 |
| **Systolic function** |  |  |  |  |  |  |  |  |
| GLS | -0.14 | 0.060 | -0.14 | 0.063 | 0.001 | 0.992 | 0.01 | 0.908 |
| GCS | -0.16 | **0.042** | -0.17 | **0.032** | -0.12 | 0.062 | -0.13 | **0.042** |
| LVEF | -0.17 | **0.028** | -0.16 | **0.038** | -0.17 | **0.007** | -0.16 | **0.012** |
| **Diastolic function** |  |  |  |  |  |  |  |  |
| E/A | 0.23 | 0.008 | 0.18 | **0.010** | -0.05 | 0.472 | -0.07 | 0.314 |
| E/e’ | -0.12 | 0.166 | -0.05 | 0.537 | -0.06 | 0.427 | -0.04 | 0.610 |

^a^Multivariable model adjusted for age, ethnicity, smoking, BMI, and systolic BP.
E/A = early to late diastolic mitral inflow velocity ratio; E/e’ = early diastolic mitral inflow to annular velocity ratio; ECV = extracellular volume fraction; GCS = global circumferential strain; GLS = global longitudinal strain; LVEF = left ventricular ejection fraction; LVM/V = left ventricular mass to end-diastolic volume ratio.

Table S3 MPR in T2D and controls segregated by two quantitative perfusion methods

|  | 1.5T (Fermi) | | | | | 3T (Kellman) | | | | |
| --- | --- | --- | --- | --- | --- | --- | --- | --- | --- | --- |
|  | n | T2D | n | Controls | P value | n | T2D | n | Controls | P value |
| MPR | 149 | 2.91 ± 1.03 | 45 | 3.88 ± 1.17 | <0.001 | 270 | 2.88 ± 0.84 | 68 | 3.13 ± 0.83 | 0.027 |

MPR = myocardial perfusion reserve; T2D = type 2 diabetes.

# Supplemental Figures


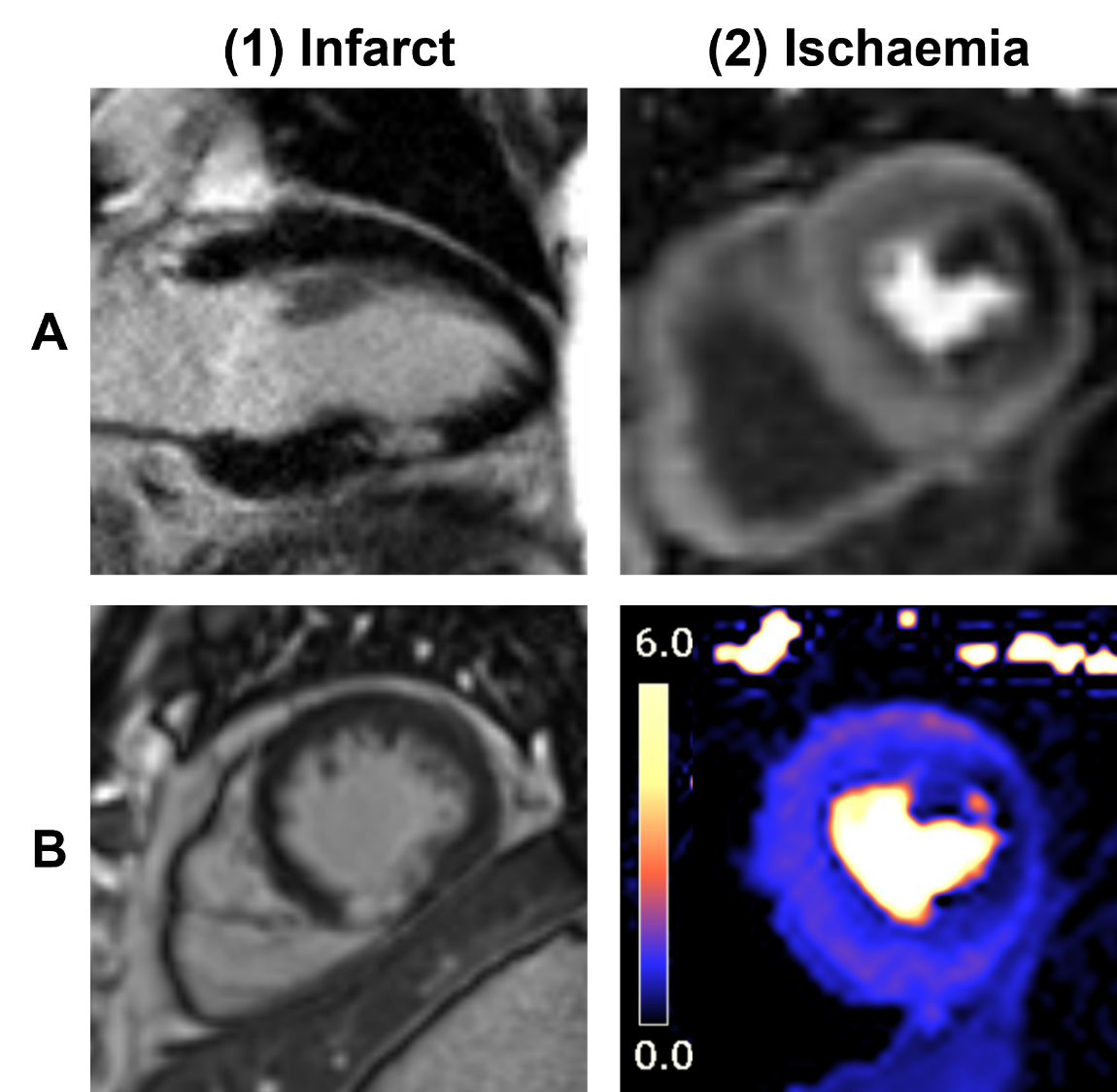


Figure S1 Representative MRI images of participants excluded due to discovery of silent infarct or ischemia. (1A and 1B) Long and short axes late gadolinium images with mid inferior segment near-transmural enhancement. (2A) First-pass and (2B) perfusion map images of the mid-ventricular short axis slice with transmural perfusion defect in the mid lateral segments
